# On sentiment recognition mechanism in Black Myth: Wukong player communication on Youtube

**DOI:** 10.3389/fpsyg.2025.1625671

**Published:** 2025-10-07

**Authors:** QinLi Tang, XueJiao Bai, Feng Gan

**Affiliations:** 1 Anhui Broadcasting Movie and Television College, Hefei, China; 2 Department of Arts, International College, Krirk University, Bangkok, Thailand; 3 School of Arts, Southeast University, Nanjing, China

**Keywords:** game communities, emotional mechanism, opinion leaders and fake engagement, cross-cultural communication, user comments and behavior analysis

## Abstract

**Introduction:**

As digital games become an important medium for global cultural dissemination, social media platforms have gradually become the primary space for players to express emotions and interact. However, systematic research on emotional expression and communication mechanisms within gaming communities is still relatively weak, particularly in cross-cultural contexts, where the impact of opinion leaders and abnormal interaction behaviors has not been thoroughly explored.

**Methods:**

Based on user comments of Black Myth: Wukong on YouTube, this study uses natural language processing (NLP) and social network analysis methods, combined with sentiment analysis and topic modeling (LDA), to analyze 7,604 comments in terms of sentiment distribution, interaction intensity, and identification of potential manipulation behaviors.

**Results:**

The results show that the majority of comments are neutral to positive in sentiment, with discussions focusing on game mechanics and cultural narratives. Some high-engagement comments display signs of opinion guidance or “water army” interference, particularly in discussions on specific topics and storylines.

**Discussion:**

This study further expands the application of social network theory and sentiment assessment theory in gaming community research, providing theoretical support and practical value for cross-cultural acceptance studies, false interaction detection, and digital community governance.

## 1 Introduction

This study investigates the emotional communication mechanisms and community interaction patterns found in audience comments on the globally popular game Black Myth: Wukong. It addresses two core questions: (1) How can we quantitatively analyze emotional expression and social interaction within player comments? (2) How can we build a multidimensional framework to detect bot-like behaviors accurately? The overarching goal is to establish theoretical links between game review data, emotional mechanisms, and community behavior dynamics.

In the context of the global digital gaming boom, Black Myth: Wukong, a domestically developed Chinese game, has seen phenomenal commercial success—over 20.5 million units sold on Steam and multiple awards at The Game Awards. However, the mechanisms behind its cultural impact and market performance remain underexplored. Prior studies present several disciplinary limitations: communication studies focus primarily on symbolic decoding (Hengbo, 2024), industrial economics on sales metrics, and social computing on user representation models (Nurrosyidah and Wang, 2025). This study adopts an interdisciplinary approach that integrates social network centrality theory, emotional appraisal frameworks, and topic modeling to provide a comprehensive analytical toolkit for examining online game communities.

While recent research has contributed valuable insights, several theoretical and technical gaps persist. For instance, Casprini et al. (2020) explored how individual passion affects game development strategies but could not explain AAA-level tensions between cultural identity and capital logic (e.g., globalized edits that dilute local narratives). Wulf et al. (2021) examined parasocial interaction on Twitch but failed to consider how platforms like YouTube—via algorithmic tags such as “cultural controversy”—may artificially inflate engagement or distort cross-cultural interpretation (e.g., Western versus Chinese readings of the Monkey King). Kim and Lee (2024) applied HBIM techniques to structured heritage data but did not address the noisy, unstructured nature of gaming comments (e.g., memes, abbreviations) or colonial semantic bias (e.g., the Western misreading of the “Golden Cudgel” as a mere weapon). Similarly, Bongers and Vallejos (2023) analyzed class metaphors in games but lacked quantification of grassroots creative resistance (e.g., MOD culture). Hawreliak and Lemieux (2020) discussed systemic oppression in games but did not offer technical strategies to counter algorithm-driven manipulation or identify inauthentic activity.

To address these gaps, this study builds on Kim and Lee (2024) critical data validation framework and introduces two key innovations:

A multimodal clustering mechanism for bot detection, adapting HBIM’s structured logic to social media analysis. Using LDA topic modeling and Casprini et al.’s (2020) passion classification, it distinguishes genuine user passion (e.g., strategy around boss fights) from strategic, bot-driven narratives (e.g., repetitive cultural controversy).

A semantic attraction model for bilingual analysis, extending Hawreliak and Lemieux (2020) multimodal framework to uncover how mechanics like the “tightening spell” evoke different cultural interpretations—Chinese players see it as a metaphor of discipline, while Western players focus on combat fluidity.

Building on this foundation, the study employs social network theory and emotional appraisal theory to analyze user comments from Black Myth: Wukong videos on YouTube. Natural language processing techniques are used to extract sentiment and topic structures, revealing emotional distribution and key discussion themes. Social network analysis maps user interactions, identifying node centrality, KOLs, and potential bots. By integrating quantitative modeling with qualitative interpretation, this research offers a systematic examination of user behavior and its underlying motivations.

## 2 Literature review: the relationship between games and media, and comment interaction patterns

With the continuous development of digital media, video games have evolved from traditional entertainment forms into a highly interactive medium and now constitute a central component of contemporary digital culture. Unlike traditional media such as film and television, which operate through one-way information transmission, video games offer immersion and interactivity, affording users a deeper sense of participation and communal experience. Yılmaz et al. (2022) argue that these media attributes position games not merely as entertainment but also as pivotal platforms for cross-cultural communication and social bonding. Cerezo-Pizarro et al. (2023) further note that games, through their integration of diverse media forms, interactivity, replayability, and embedded narratives, offer unparalleled capacities for cultural expression and real-world engagement.

Within this media ecology, user comments have emerged as a key conduit linking players to game communities. Far beyond expressions of individual opinion, these comments now operate as affective nodes that bind digital communities. Naab (2022) shows that the emotional tone of comments significantly shapes other users’ experiences and decisions, while Chandrasekaran et al. (2025) demonstrate that social actions like likes and replies promote emotional resonance and help stabilize virtual community structures.

Building on this foundation, this study adopts the Appraisal Theory of Emotion (Hu et al., 2023) as its core framework for analyzing emotional expression. This theory asserts that emotional responses stem not from stimuli per se but from individuals’ subjective evaluations of meaning. In the gaming context, players’ emotional reactions to narrative, mechanics, or community discourse are shaped by rapid cognitive judgments—e.g., whether the experience aligns with expectations or generates personal resonance. This cognitive-emotional loop underlies sentiment formation and contributes to the social energy sustaining digital community participation.

To investigate how these emotional responses evolve into structural community patterns, this study also introduces Social Network Theory (Song et al., 2023) as the basis for modeling interaction. According to this framework, users form dynamic relational networks through actions like likes, replies, and mentions, where certain high-centrality users (e.g., KOLs or “social avatars”) exhibit stronger influence in shaping emotional climates and group behavior. By integrating sentiment intensity with network metrics, the study identifies not only opinion leaders but also abnormal diffusion nodes.

While prior literature provides valuable groundwork, it often stops at summarizing emotional dynamics or mapping visible influence, without addressing how emotional flows evolve across time and structure—especially in the presence of manipulation. For example, Ping et al. (2023) note that KOLs guide community discourse through emotional engagement, while Lankes and Stöckl (2023) warn that AI-generated comments blur the boundaries between authentic and inauthentic actors. However, empirical analysis of how these mechanisms operate *in situ*—particularly under platform algorithmic influence—remains limited.

This study responds to those gaps by proposing a tripartite analytical framework—emotional appraisal, social diffusion, and group clustering—operationalized via sentiment analysis, LDA topic modeling, and social network analysis. In contrast to research that primarily classifies emotions or tracks influencers, we explore how fragmented emotional responses converge into collective identity within a dynamic and sometimes manipulated environment.

In doing so, we address key limitations in existing methodologies. Common sentiment lexicons often fail to capture sarcasm, irony, or culturally embedded meaning in comments. Similarly, centrality indicators alone cannot detect covert, low-visibility behaviors like organized astroturfing. To overcome this, we introduce multidimensional indicators, including like-reply trajectories, posting frequency patterns, and network flow metrics, to trace how emotional expressions are amplified, modulated, and consolidated into communal attitudes.

This approach allows us to model the “dynamic evolution” of emotion within digital networks, acknowledging both platform-level algorithmic shaping and user-level agency. Given the shortcomings of static community detection models in representing fluid group behaviors, we adopt a comparative methodological lens to evaluate clustering logics under real-world constraints.

Ultimately, this study reframes the concept of “audience” into that of a networked digital community, emphasizing how platforms like YouTube serve as emotionally charged spaces of cultural production. As Antonio et al. (2024) suggests, understanding digital feedback mechanisms requires not only decoding what is said but also mapping how it circulates, where it flows, and the meaning it accumulates along the way. By investigating emotional generation and social structuring in tandem, we aim to clarify the boundaries between authentic communities and coordinated manipulative behaviors—offering both theoretical advancement and practical implications for digital game design and global cultural discourse.

## 3 Constructing the analysis framework for Black Myth: Wukong user feedback

### 3.1 Quantifying emotional content in unstructured comments

This study uses a combination of sentiment quantification and clustering analysis to systematically analyze user feedback on Black Myth: Wukong from both qualitative and quantitative dimensions. The clustering analysis uses the unsupervised learning K-means algorithm to identify potential audience groups and their emotional structures behind game reviews, which is particularly suitable for research subjects whose target user characteristics are not well-defined. To extract potential themes in review texts, this study uses Latent Dirichlet Allocation (LDA) modeling, borrowing from Lee et al.’s (2021) application of LDA in social media and sustainability studies to identify emotional differences and interaction trends among topics. To ensure the accuracy and stability of the analysis results, the study collects 7,604 reviews(data collection date: July 2025), which meets the sample size requirements set by Blei et al. (2003) for topic models, i.e., the greater the number of topics, the larger the sample size required.

For sentiment analysis, referring to Lau et al.’s (2024) approach in media literacy emotional profiling studies, this paper uses a five-dimensional scale for sentiment evaluation: 1 for extreme dissatisfaction, 5 for very satisfied, and 3 for neutral,to strengthen the approach’s ability to capture emotional extremes. At the same time, to more accurately reflect audience behavior intensity, this study introduces likes and replies as weighted indicators for filtering threshold. Based on previous research that identified interaction behavior differences (Lau et al., 2024), replies are assigned higher weights as they represent deeper user expression and opinion interaction, whereas likes are seen as shallow emotional recognition. Therefore, comments are analyzed by combining the number of replies and likes, resulting in Table 1.

**Table 1:** Comment reply analysis table.

Indicator	Median (50%)	95th percentile	99th Percentile	Maximum value
Reply Count	0	1	10	725
Thumbs Up Count	0	35	510	59,000

By examining the descriptive statistics of reply count and thumbs up count in the data, it was found that the 99th percentile of reply count is 10, and the 99th percentile of thumbs up count is approximately 510. To ensure that the selected comments belong to the top subset with the highest heat and to facilitate practical operations, the filtering threshold is set as follows: reply count ≥ 10 and thumbs up count ≥ 500. This threshold is close to or slightly below the 99th percentile, representing the comments with the highest heat and influence in the data. Based on this criterion, users with higher reply counts and thumbs up are selected as Key Opinion Leaders (KOLs). These KOLs’ comments not only receive high recognition (high thumbs up) but also demonstrate strong influence and distinction in reply discussions. The specific KOL topic clustering results are detailed in Table 2.

**Table 2:** KOL clustering table.

Author	Content	Reply	Thumbs_up	Sentiment
01	People with no ps5,good pc or just cannot afford to buy the game yet, we come together here	542	15,000	2.2
02	Full game + true ending you sir are the chosen one	26	7,300	3.2
03	Still make me go crazy that they visited and studied the actual mythology and character so well the animation could not be more accurate.	44	3,200	3.15
04	2 h and 56 min in and i’m absolutely convinced that this game deserves game of the year,i Hope It sells well	38	1,600	2.6
05	On the surface, Journey to the West is about defeating demons and monsters, but at a deeper level, it talks about the struggles and transactions between ruling, being ruled, and political factions, as well as the resistance of the Great Sage (the people). This novel, written in the 16th century, describes the social rights rules of that time, but it is still valid for today’s Chinese society, and even for the interpretation of social rules in most countries, it is still very effective.	19	511	3.61
06	3 things that made this game will be my number 1 favourite for long long time: 1. The storytelling was so good to the point where i could not wait to watch the next cutscenes. 2. The character buildings are very captivating. I’m now fans of bajie, that pig who reminds me of that friend who always being a jerk infront of us, but will do everything to help when we are in trouble. 3. The graphic is top notch. Just look at the grassland where violet spider died. When the wind blows, we can see the flower powder, wilted leaves, their long hair, all move in the same direction. It was a cinema quality that really close to pixel studio. This game is amazing. Really a rare one. Worth every cents of the price	15	978	3.26
07	Fun fact: At the start of the boss fight, Erlang, is a true chinese god, that is wu kong’s rival. Wukong is said to have 72 unique powers and Erlang have 73 unique powers so technical erlang should be more powerful. Much like Byakuya from bleach, Erlang is often cold and emotionless. Have highest standards of conduct and expects the same from those around him. Which is why he cannot stand Wukong’s mischievous act	11	1,000	3.33
08	The opening was overwhelming. Felt like watching an anime. The trailer and pre-release stood upto our expectations. Magnificent.	15	1,100	4.5
09	Feels like I’ve waited for this game for half a decade now.	73	6,900	2.43
10	Gambit: “Do you know how long I have been waiting for this?”	25	4,500	2.9

To systematically address the research objectives, the study proposes the following two hypotheses:

*H1*: There is a positive correlation between the emotional tendency of comments and the interaction heat they receive (number of likes and replies), and different emotional tendencies exhibit significant differences in topic clustering distributions. This hypothesis will test whether emotional expression influences user interaction behaviors and explore how emotional characteristics manifest in topic-level classification structures.

*H2*: Game comments display dual differentiation characteristics of emotion and behavior in community clustering, with concentrated areas of extreme emotions and high interaction heat possibly indicating abnormal behavior patterns (such as bots).

### 3.2 Structure and characteristics of audience groups

This study selects YouTube comments as the sole data source for analyzing audience feedback on Black Myth: Wukong, primarily due to YouTube’s rich interaction structure and content depth. As the world’s largest video-sharing platform, YouTube provides a massive volume of user-generated data, often accompanied by detailed opinions, emotional expressions, and diverse cultural perspectives. Unlike platforms such as Twitter, where character limitations restrict depth, YouTube allows users to elaborate more fully on their reactions. Moreover, the global user base ensures that feedback spans different linguistic, regional, and demographic groups, making it an ideal platform for observing emotional and behavioral patterns in a cross-cultural context.

That said, the exclusive use of YouTube introduces certain limitations. User behavior varies significantly across platforms: Twitter tends to reflect quick, impulsive reactions, while YouTube fosters more sustained engagement. As a result, focusing only on YouTube may obscure other interaction modes or emotional dynamics. The absence of comparative cross-platform data also introduces potential sampling bias, as the study cannot capture the full emotional spectrum or interactional variation among audiences on other platforms. Consequently, the findings primarily reflect behaviors within video-based interactive environments, rather than the entire gaming audience.

To ensure data quality and analytical rigor, this study used web scraping tools to collect YouTube comment data related to Black Myth: Wukong. Given that most comments were in English (with a small portion in Arabic and other languages), English was selected as the baseline for semantic analysis. Key variables—such as reply count, like count, and sentiment score—were extracted for each comment.

The following text preprocessing steps were applied to improve analytical precision:

Removal of special characters, numbers, and stopwords.
Text normalization (lowercasing and spelling correction).
Stemming and lemmatization to reduce semantic redundancy.
Tokenization to segment text into analyzable units.

These steps ensured cleaner inputs for the subsequent clustering and sentiment modeling stages.

Based on the cleaned data, the study employed Natural Language Processing (NLP) to conduct sentiment analysis, assigning each comment a sentiment score on a 1-to-5 scale:

1 = strongly negative.
2 = slightly negative.
3 = neutral.
4 = slightly positiv.
5 = strongly positive.

To explore user clustering and interaction patterns, K-means clustering was used. The optimal number of clusters (K) was determined by combining the Elbow Method (which identifies the inflection point in SSE decline) and the Silhouette Score (which evaluates intra-cluster cohesion and inter-cluster separation). Topic modeling (via LDA) was also conducted to identify thematic structures across comment clusters.

To assess the quality of clustering, the study reports both coherence scores (semantic consistency within clusters) and perplexity scores (textual compactness and interpretability). Lower perplexity and higher coherence were used as indicators of successful clustering.

For behavioral analysis, the study introduces several operational definitions:

Bot-like behavior is defined by a combination of high sentiment uniformity (low standard deviation), repeated cross-topic activity, and templated or formulaic comment structures.

Key opinion leaders (KOLs) are identified based on network centrality measures (e.g., number of replies, likes, and in-degree influence) in the social graph.

To validate the accuracy of classifications (e.g., clustering results or bot detection), a manual coding procedure was used. A subset of 200 comments was independently reviewed and labeled by two trained coders. Inter-coder reliability was assessed using Cohen’s Kappa (*κ* = 0.81), indicating strong agreement and validating the machine-assisted categorization results.

Together, these combined approaches—text mining, clustering, sentiment scoring, and social network analysis—form an integrated framework that enables the study to comprehensively examine emotional expression, interaction behavior, and abnormal patterns such as synthetic engagement or manipulation.

**Figure 1:**
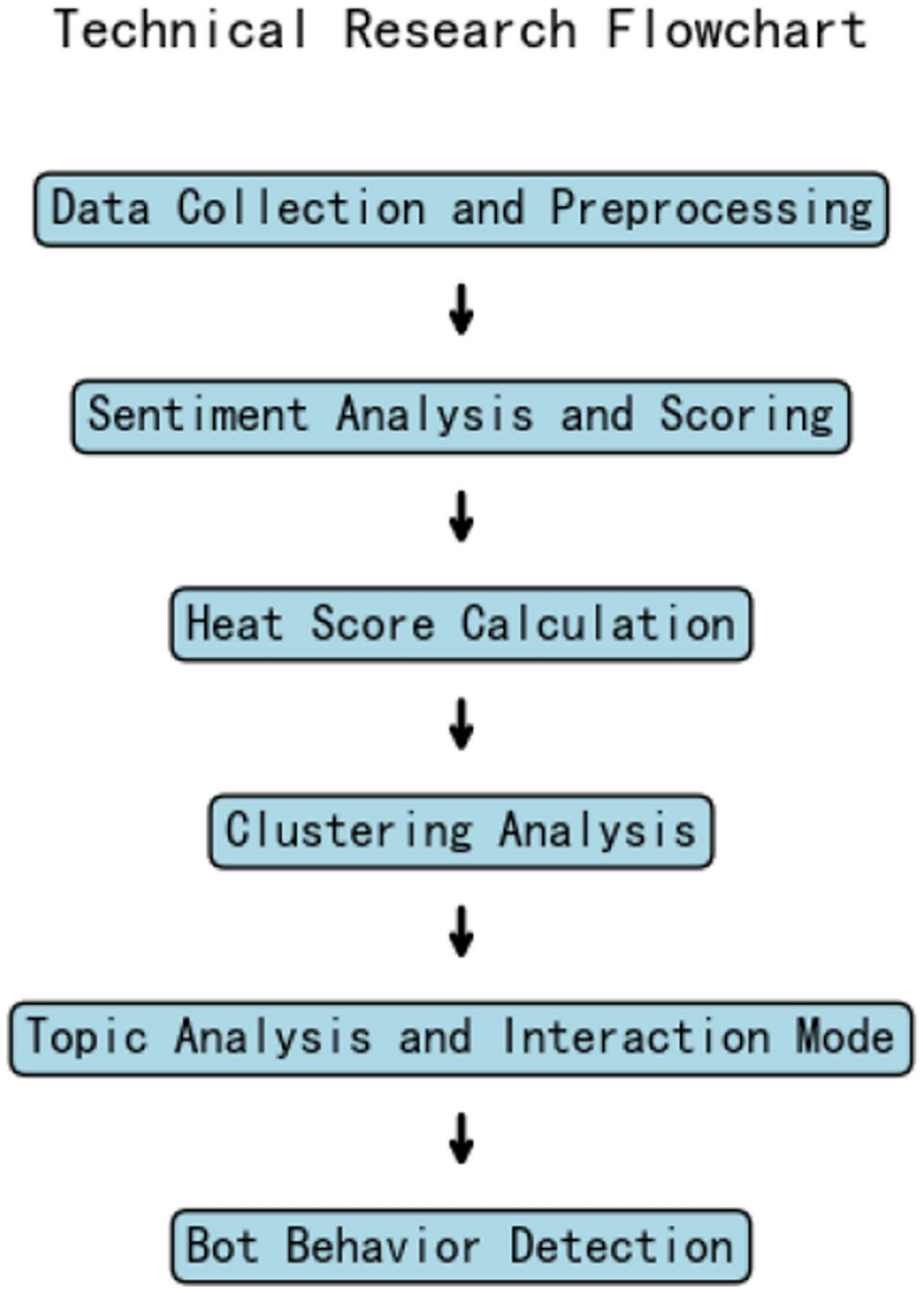
Technology roadmap.

Figure 1 illustrates the technical roadmap. Based on this figure, the research path follows a process where, after completing the preliminary data cleaning, sentiment analysis and rating are performed first. This step not only provides a general understanding of the audience’s emotional preferences but also serves as an important reference for subsequent heat analysis, interaction pattern analysis, and clustering analysis. It helps further explore the relationship between sentiment and interaction. The process of generating sentiment scores in this study is as follows:

The analysis of all the data was conducted following the process outlined in Table 3, and line charts were created as shown below (the values in the chart are rounded to integers, with decimal points ignored).

**Table 3:** Sentiment analysis table.

Sentiment score	Cleaned comment	Explanation
1	The gameplay isn boring what?	Strongly negative, contains denial, criticism, or emotional language.
2	Very difficult game	Slightly negative, contains mild complaints or doubts.
3	Damn I finished the guangzi and then do not know where to go here you stand already finished the game	Emotionally neutral, objective description without clear sentiment.
4	This comment made me realize how blessed i am i have to stop complaining about dum sht	Slightly positive, expresses approval, support, or positive comments.
5	If you can. I think people will love it	Strongly positive, expresses strong liking or praise.

**Figure 2:**
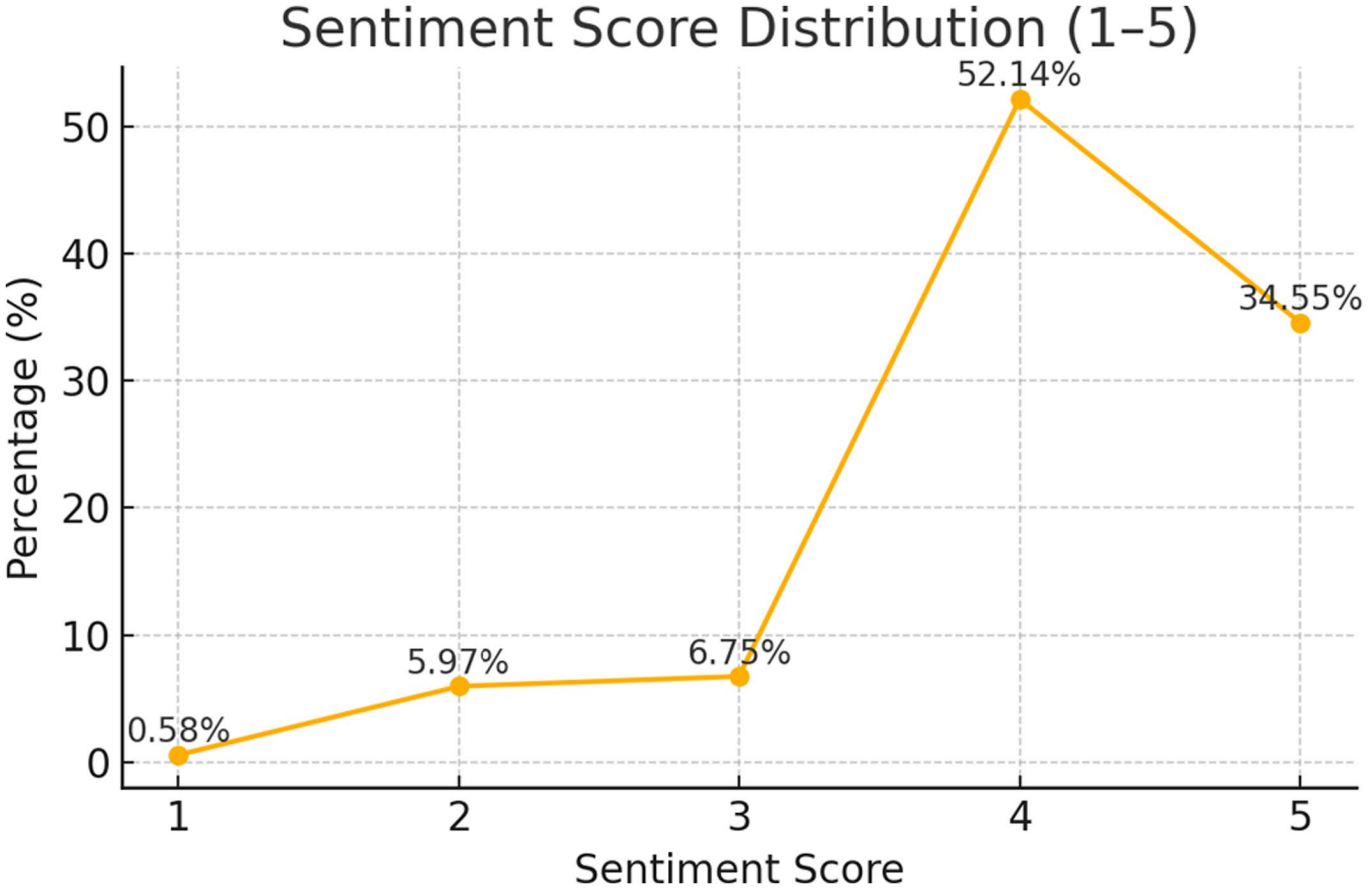
Sentiment score distribution figure.

From Figure 2, it can be seen that, based on the sentiment score distribution and the annotations on the line chart, overall, a score of 4 (slightly positive) accounts for the highest proportion, reaching 52.14%. This indicates that most users have a mildly positive attitude, expressing recognition but without strong emotional intensity. The next highest proportion is score 5 (strongly positive), which accounts for 34.55%. More than a third of the comments express clear liking or praise, suggesting that the topic or content is highly appealing. Scores 1–3 together account for less than 15%, with score 3 (neutral) at 6.75%, score 2 (slightly negative) at 5.97%, and score 1 (strongly negative) at only 0.58%. Negative comments make up a very small proportion, and neutral comments are also relatively few, indicating that negative feedback is very limited, with users primarily focusing on positive emotions. In summary, the public opinion direction is highly positive, and the overall community atmosphere is optimistic. Furthermore, by further combining interaction data, such as replies and likes, we can gain deeper insights into which emotionally inclined comments are more likely to spark discussions and gain support. This helps in better understanding user interaction patterns and effectively analyzing improvements for negative feedback.

**Figure 3:**
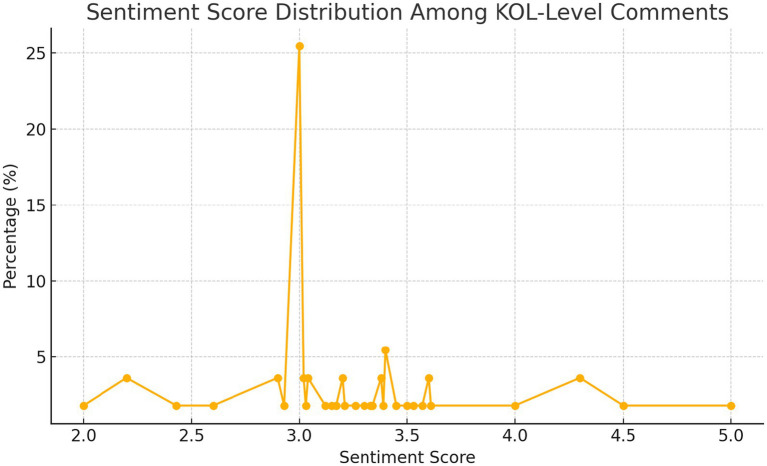
Sentiment score distribution among KOL-level comments.

Further analysis of sentiment scores and interaction data based on Figure 3 reveals that comments meeting the high reply and high thumbs up thresholds primarily have sentiment scores around 3, showing a neutral-to-positive tendency. Extreme negative (score 1) or extreme positive (score 5) comments make up a very small proportion of high-engagement comments, indicating that, in actual discussions, emotionally balanced, rational, or mildly positive comments are more likely to gain user approval and generate widespread discussion. This also suggests that, in brand communication or content management, comments with moderate emotional expression and a neutral-to-positive tone are more characteristic of KOLs (Key Opinion Leaders) and have a stronger effect on guiding public opinion.

Through optimized clustering analysis, all KOLs meeting the filtering criteria from Table 1 were selected, totaling 55. These 55 were divided into five main topics. Based on the number of keywords and subtopics for each topic, comments with similar topic keywords were grouped together, forming Table 4.

**Table 4:** Topic clustering table.

Topic tag	Keywords	Number of subtopics
0	Akira, ball, dragon, toriyama, anime, environment, fights, mythology, characters, expectations.	11
1	Boy, ending, energy, game, english, edit, emotionless, cloud, weapon, lore.	25
2	Aaa, forgotten, fromsoft, game, critics, diversity, generations, meaningful, history, phenomenal.	8
3	Buddha, earned, fighting, endings, victorious, thank, positive, laugh, remember, school.	3
4	Background, chinese, goku, japanese, myth, wukong, god, original, powers, relics.	8

Table 4 divides the comments into five topics, covering different areas of interest. Topic 0 is related to the Dragon Ball anime, involving discussions about various characters, scenes, and narratives. Topic 1 focuses on the game’s ending and storyline development, discussing different types of games, player feedback, and energy systems, among other topics. Topic 2 is closely associated with classic action games like Dark Souls, with the focus of the discussion on game design, difficulty, and challenges. Topic 3 is more philosophical, involving discussions about Buddhism and cultural background, particularly focusing on Buddhist perspectives and life philosophy. Topic 4 focuses on the cultural differences between China and Japan, analyzing cultural elements and character backgrounds in Dragon Ball. Each topic presents distinct discussion characteristics and areas of focus.

From the perspective of social network theory, the formation and discussion of these topics may reflect the social interaction patterns among commentators. The extensive discussions in Topic 0 and Topic 1 suggest that these may be central topics in larger communities, attracting more interaction and information exchange. In contrast, smaller subtopics (such as Topic 3 and Topic 4) may be concentrated in niche communities, where the discussions are more in-depth but with lower participation. Information dissemination in social networks may have played an important role in the formation of these topics, with central topics often attracting more attention through widespread spread and interaction, while niche topics may gain attention through deep exchanges within specific groups.

To further illustrate, 10 comments that meet the filtering threshold criteria from Table 1 are selected as examples. There are a total of 55 comments that meet the above criteria, but due to space limitations, only 10 are selected for demonstration.

In the above comments that meet the threshold, the relationship between these comments and KOLs (Key Opinion Leaders) can be analyzed from multiple perspectives. Firstly, most of the comments have a neutral or positive sentiment score, reflecting the high attention and enthusiasm that players have for Black Myth: Wukong. For example, comment 08 states, “The opening was overwhelming, felt like watching an anime,” which shows high expectations and praise for the game, with a sentiment score of 4.5, indicating a strong emotional influence. Comment 07 mentions, “Erlang is Wukong’s rival with 73 unique skills,” which not only demonstrates a deep understanding of the character’s background but also explains the game’s character setting through comparison. Such comments have educational and guiding qualities, helping other players better understand the game’s world view, aligning with the characteristics of KOL behavior.

Secondly, comments 01 and 09 show emotional buildup and anticipation for the game over time. The users express their emotions and interact with other players. For example, comment 01 states, “People who do not have PS5 or cannot afford the game are gathered here,” showing a sense of community and resonating with many players’ feelings. Such comments tend to generate higher interaction and responses, which help spread the topic. These comments generally have high viral potential because they not only reflect individual emotions but also spark resonance and discussion among other players. Especially under the influence of KOLs, emotional and interactive dissemination becomes more rapid.

Moreover, comment 05 demonstrates an understanding of the deeper social and political metaphors in Journey to the West, showing the user’s reflection on the cultural background behind the game. This type of comment provides additional cultural perspectives and insights, fulfilling the guiding role of KOLs in the community. Through such comments, the user not only conveys high praise for the game but also stimulates other players to think about the deeper meanings of the game, enhancing their identification with it. Comment 06 offers a detailed analysis of the game’s plot, character development, and visual effects, showing a profound understanding and high appreciation of various aspects of the game. Such detailed analysis not only provides valuable feedback but also guides other players to focus on these highlights within the game.

Finally, the language style of the comments is often direct and emotionally charged. For example, comment 03 mentions, “The animation was so precise, it drove me crazy,” and this direct emotional expression makes the comment highly impactful. Such comments quickly capture the audience’s attention and encourage emotional resonance, making them a powerful tool for KOLs in spreading influence on the platform. In summary, these comments not only reflect the enthusiasm and in-depth discussion players have for Black Myth: Wukong but also demonstrate a high degree of alignment with KOL behavior. Through these comments, KOLs help expand the game’s community effect and spread topics by guiding emotions, providing in-depth analysis, and fostering interactive feedback, which actively promotes the game’s influence and reputation.

Having previously explored the emotional distribution of the game audience, topic clustering, and the role of KOLs, the next step is to investigate whether there exists a community distribution within the game audience. To do so, cluster analysis is employed on the game audience data. In this study, the K-means method is used for clustering, where “K” represents the number of clusters. To determine the optimal value of K, both the Elbow Method and Silhouette Score are applied.

The Elbow Method involves observing the trend of the sum of squared errors (SSE) within the clusters, choosing the “elbow point” where the rate of SSE decline significantly slows (Ketchen and Shook, 1996). The Silhouette Score evaluates the density and separation of the clusters, selecting the K value that maximizes the silhouette score (Rousseeuw, 1987). By combining these two methods, the optimal clustering effect is ensured.

**Figure 4:**
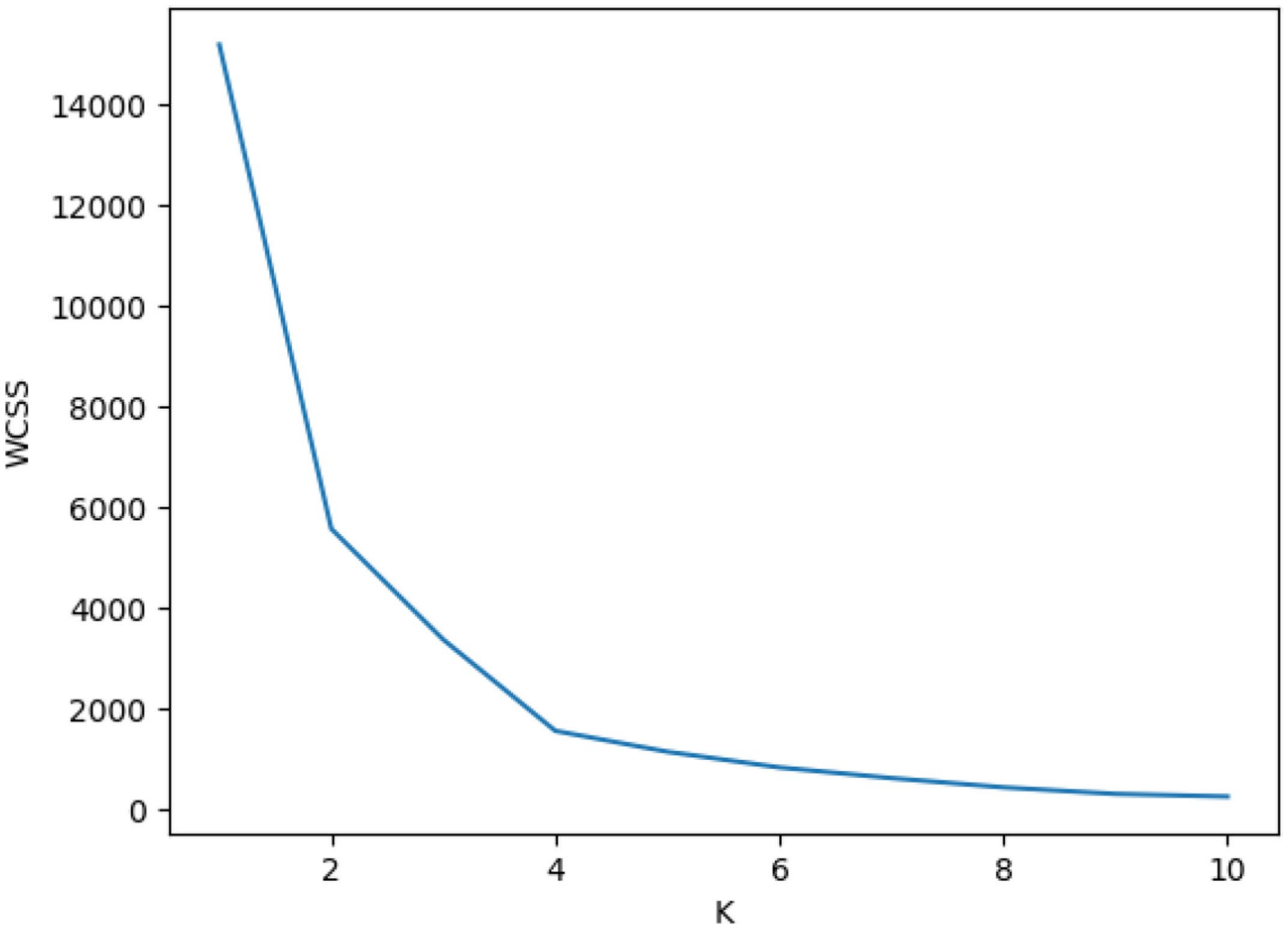
Elbow method figure.

**Figure 5:**
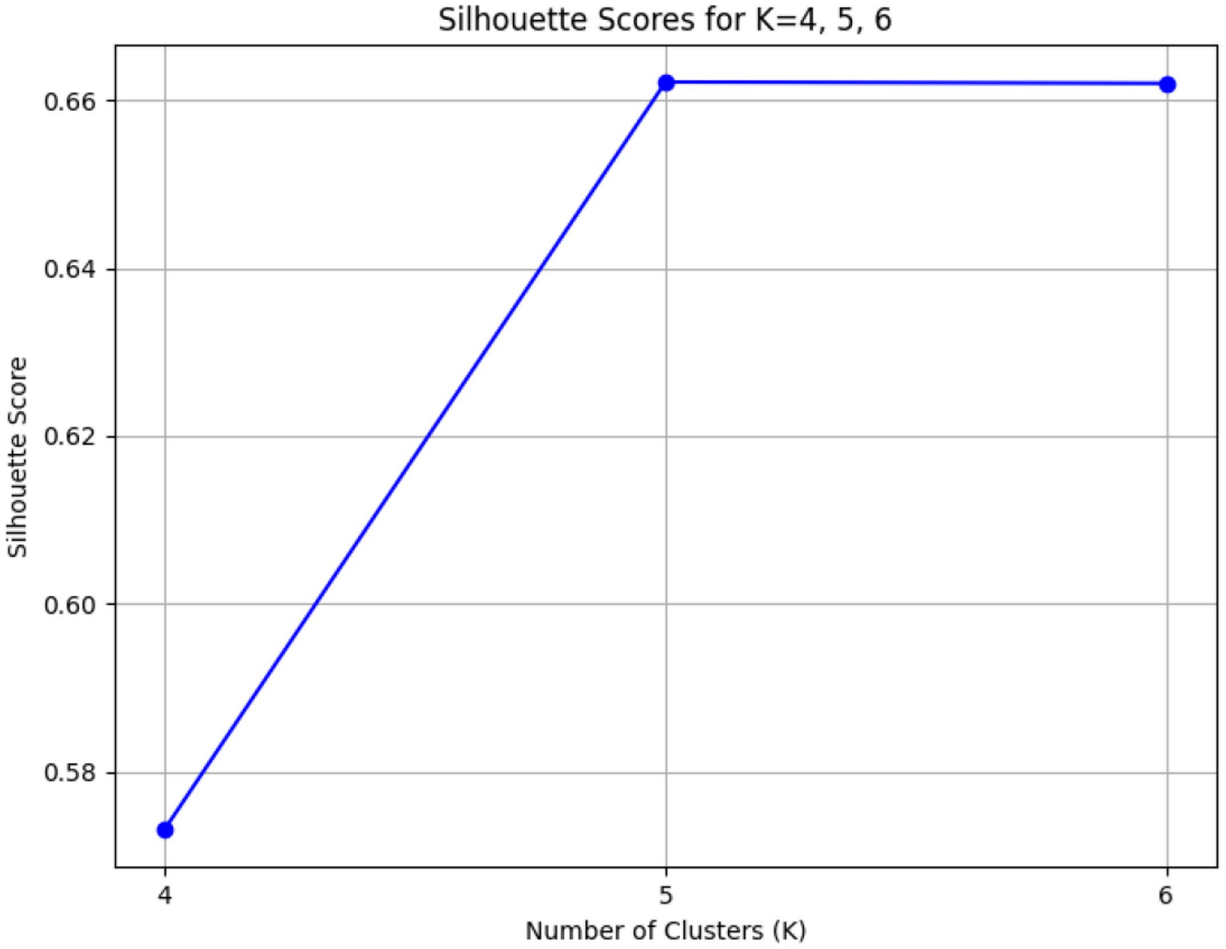
Silhouette scores figure.

The process begins with the Elbow Method:

Based on the elbow method analysis in Figure 4, the SSE decreases rapidly as the value of K increases, but the rate of decrease levels off between K values of 4 and 6, indicating that a balance has been achieved between reducing SSE and preventing overfitting. To further confirm the optimal K value, the silhouette coefficient (Figure 5) was used to evaluate the clustering quality for different K values. By measuring the separation and cohesion of the clusters, the optimal number of clusters was ultimately determined.

The higher the value of the Silhouette Score, the better the clustering effect. Among these results, the Silhouette Score for K = 5 (0.662125) is the highest, slightly higher than that for K = 6 (0.661962), while the score for K = 4 is relatively lower (0.573204). Therefore, K = 5 is the most suitable number of clusters, as it has the highest Silhouette Score, indicating the best clustering performance.

**Figure 6:**
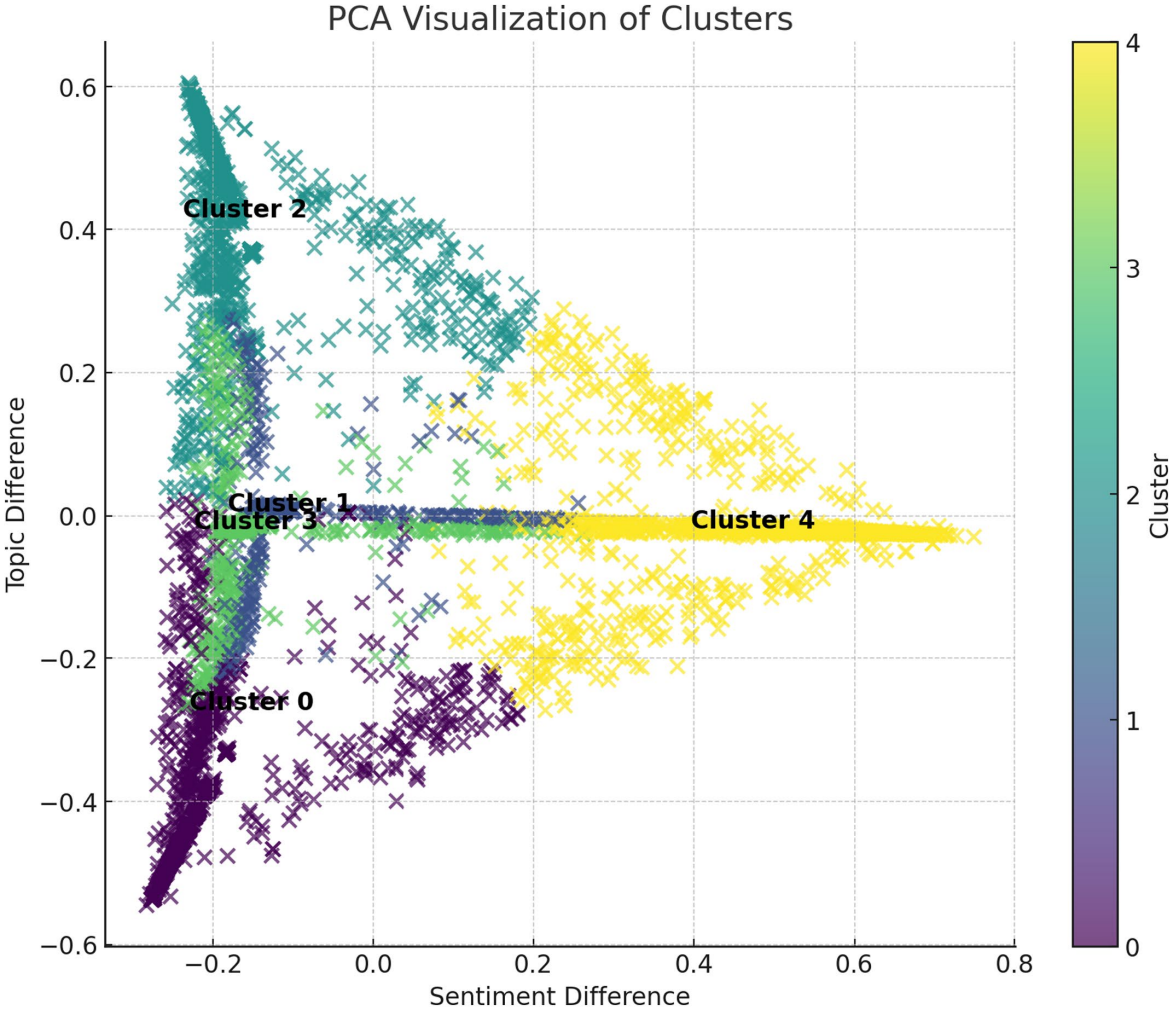
Game audience clustering figure.

Below is the feature and trend analysis for each cluster:

Based on Figure 6, coherence and perplexity are calculated. Coherence and perplexity were originally applied in natural language understanding tasks to evaluate sentence fluency and the NLTF model’s ability to predict generated text. In clustering algorithms, the relationship between topics is typically measured by calculating the similarity of content within each topic, using the correlation between the comment content and the topic as an indicator of coherence. Additionally, the distance between the points in the scatter plot is set as a predictive measure to further assess the structure and consistency of the text (Blei et al., 2003).

Based on the content of Figure 6 and Table 5, and combined with sentiment evaluation theory and social network theory, the topics exhibit different emotional tendencies and social interaction characteristics. Cluster 0 (emotion and personal relationships) and Cluster 3 (mourning and sadness) involve negative emotions, discussing themes such as loss and the death of loved ones. They show high coherence (0.9925 and 0.9928, respectively), indicating that the text is consistent and fluid, with strong emotional continuity. Additionally, low perplexity (0.6397 and 0.5779, respectively) indicates tight clustering, with the text being semantically focused. These topics can provoke strong emotional resonance in social networks, with users discussing these negative emotions, forming a social circle of emotional support.

**Table 5:** Cluster analysis chart.

Topic clustering	Color	Number of comments	Content summary	Distribution description in the Figure	Perplexity	Coherence
Cluster 0	Dark Purple	Medium number	The topic revolves around emotions, loss, sadness, and personal relationships, involving words like “sorry,” “loss,” “brother,” etc.	Located on the left side of the figure, more concentrated, indicating negative emotional differences.	0.639710183	0.992522629
Cluster 1	Dark Blue	Larger number	The topic is mainly game-related, discussing gameplay, the quality of the game, money, etc., involving words like “game,” “play,” “money.”	Located at the top right of the figure, with a more positive theme, representing positive discussions about entertainment and games.	0.569589512	0.992900158
Cluster 2	Cyan	Larger number	Mainly related to combat in the game, discussing battle, challenges, etc., involving words like “boss,” “fight,” “beat.”	Located at the bottom right of the figure, representing combat-related content, showing a higher level of confrontation.	0.584604662	0.99250964
Cluster 3	Green	Fewer number	Involves emotional and memorial content, discussing sadness, loss, and remembrance, involving words like “condolences,” “rest,” “peace.”	Located at the bottom left of the figure, focusing on emotionally negative topics.	0.577992483	0.992756122
Cluster 4	Yellow	Medium number	Related to game characters or mythology, especially discussions about “Wukong” and similar characters, involving words like “wukong,” “goku,” “god.”	Located on the right side of the figure, with the theme revolving around characters in the game, with a mythological touch.	0.5734016895287439	0.9923870210059774

In contrast, Cluster 1 (game-related) and Cluster 2 (combat-related) discuss more positive content, focusing on gameplay and combat challenges. They have lower perplexity (0.5696 and 0.5846, respectively), which further suggests tight clustering, with the content being consistent and fluid (coherence scores of 0.9929 and 0.9925, respectively). These topics foster active discussion groups in social networks, particularly through shared interests that promote information exchange and interaction. Cluster 4 (characters and mythology) centers around virtual characters and mythology, including characters like “Wukong.” Although the number of comments is moderate, the high coherence (0.9924) and low perplexity (0.5734) indicate that the content is consistent and the clustering effect is good. This topic can spark heated discussions about virtual characters, promoting cultural dissemination and the establishment of interest-based groups.

Overall, negative emotional topics drive social interaction through emotional resonance, while positive emotional topics stimulate group communication through shared interests. Additionally, high coherence and low perplexity help enhance the topic’s dissemination and social connection, making the text more engaging and promoting the flow of information and interaction in social networks.

According to Table 6, Topic 2 (Combat-related) and Topic 1 (Game-related) have the most KOLs that meet the criteria, with greater emotional dispersion, which may indicate that discussions on these topics are more intense or controversial. In contrast, the emotions in Topic 3 (Emotional & Condolences) are very consistent, and the number of KOLs meeting the selection criteria is relatively small, which may suggest that the comments on this topic are more peaceful, with fewer active participants in the discussion. Topic 4 (Game Characters) has a higher average sentiment, but with greater emotional fluctuations, which may reflect the extreme emotions players have toward game characters, showing more intense emotional responses.

**Table 6:** Sentiment distribution table for each cluster.

Cluster	Mean sentiment	Standard deviation	Minimum sentiment	Maximum sentiment	Median sentiment	KOL_count
Cluster 0	3.183636363636363	0.396970344667526	2.43	4	3.12	11
Cluster 1	3.065	0.5056412531855733	2	4.3	3	14
Cluster 2	3.329230769230769	0.5962027275245327	2.2	4.5	3.2	13
Cluster 3	3.2725	0.19517086531208147	3.04	3.5	3.275	4
Cluster 4	3.276923076923077	0.6228614695551573	2.2	5	3.2	13

## 4 Cluster behavior identification and ‘water army’ detection analysis based on sentiment evaluation and social network theory”

**Figure 7:**
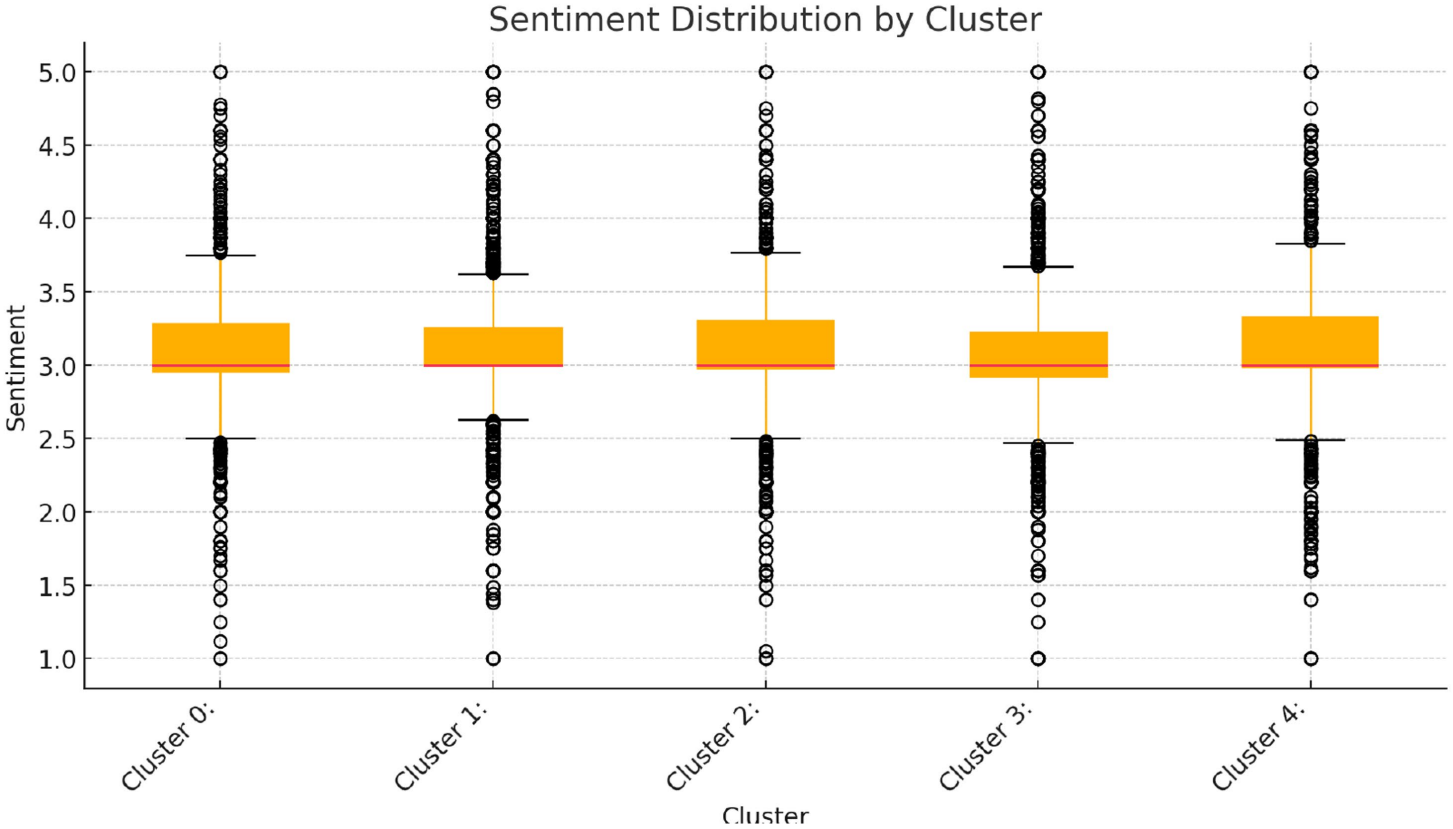
Sentiment distribution by cluster.

Based on the analysis of Table 6 and Figures 6, 7, the following conclusions can be drawn:

From the perspective of sentiment analysis, Cluster 0 shows a relatively consistent emotional response, with a mean of 3.18 and a standard deviation of 0.40, indicating that the emotional fluctuations within this group are small, and the emotional distribution is highly concentrated, reflecting emotional consistency. Cluster 1 exhibits greater emotional fluctuation, with a mean of 3.07 and a standard deviation of 0.51, indicating that the emotional responses of this group have high variability, possibly involving multiple forms of emotional expression, suggesting significant individual differences in emotional feedback. Cluster 2 shows even greater emotional fluctuations, with a mean of 3.33 and a standard deviation of 0.60, suggesting that this group’s emotional reactions may be more extreme, with significant emotional differences. In contrast, Cluster 3 shows highly consistent emotional feedback, with a mean of 3.27 and a standard deviation of only 0.20, indicating that emotional fluctuations within this group are small, and members’ emotional responses tend to be aligned, possibly forming a strong collective emotional resonance. Cluster 4, although its mean is 3.28, which is similar to other groups, has a significantly higher standard deviation of 0.62, indicating that this group has larger emotional fluctuations and more dispersed emotional feedback (Table 7).

**Table 7:** Comparison of Chinese and English Website Comments.

Chinese comments on Bilibili	English comments on YouTube
“Immortality? The Three Realms and Six Paths are destroyed by these four words, aren’t they?” Wow. this sentence is simply amazing.	“It’s No wonder a bunch of characters were inspired by This Legend.been in love with his lore ever since I was young.”
“This game is not simple, it truly has ‘Dao’ (the way).”	“Monkey was like: ‘There is too many of him, and he has a cloud, where is my cloud at?’”
“To fight against Heaven, it’s not about becoming the Great Sage, but surpassing the Great Sage. That’s why Erlang Shen helps the fated person break free from the golden hoop. This is the key to breaking the situation.”	“It has the ‘I taught you everything you know, not EVERYTHING I KNOW’ energy to it.”
“Yang Jian himself split Peach Mountain with an axe to save his mother. He is the prototype for the story of Chenxiang splitting Mount Hua.”	“That teleport kick combo is straight out of DBZ, as if Wukong saying ‘you might share my name, but I’m the original’”
“Black Myth: Wukong shows that unless you wear this ‘Best of the Year’ cassock, how would all beings know that I am the excellent game? Now there’s a bunch of people out there cursing Black Myth, and they are not asking for anything else. They’re just like Huangmei, wanting to win. Jin Chanzi does not care at all because he knows that what Huangmei is doing is a ruthless attempt to win. Even if it’s not like that, he will force it into a win. He does not even want to deal with it, and those people are the same way [watching the drama].”	“The fact that he gives you time to recover and also gives your weapon back to make you try again is a thing of a master.”

In the analysis of the number of KOLs, Cluster 1 and Cluster 2 have the largest numbers of KOLs, with 14 and 13, respectively. This may be related to their more complex discussion topics and higher levels of activity, leading to more diverse and fluctuating emotional feedback. Cluster 3 has the fewest KOLs, only 4, and its emotional feedback is more consistent, reflecting that this group’s discussions are stable and its members are less involved in broad public opinion hot spots. Cluster 0 and Cluster 4 have relatively fewer KOLs, with 11 and 13 respectively, and although their emotional responses are more consistent or fluctuate greatly, they still suggest that these groups may exhibit some emotional bias, warranting further attention.

Combined with the PCA plot analysis, Cluster 4 shows greater emotional differences, and this group’s distribution in the PCA plot is close to the horizontal axis (emotional difference) near 0, indicating that its emotional responses are more consistent, and the emotional reactions are concentrated across multiple topics. This aligns with the characteristic of “water army” behavior, where uniform emotional feedback is exerted across various topics to influence public opinion. Cluster 0 shows smaller emotional fluctuations, and with fewer KOLs, it indicates that this group’s interactions are relatively stable and emotional responses are consistent, thus ruling out the possibility of “water army” behavior. Cluster 1 and Cluster 2 exhibit greater emotional differences and higher KOL numbers, suggesting that these groups’ emotional feedback is diverse and actively involved in trending topics, which is not characteristic of “water army” behavior but rather represents normal social interaction. Cluster 3’s emotional responses are stable with a smaller standard deviation, and with fewer KOLs, it indicates that this group’s emotional feedback is consistent, and its behavior aligns with general social interactions rather than typical “water army” behavior.

In conclusion, Cluster 4 is most likely to exhibit “water army” behavior, as it shows highly consistent emotional responses, smaller emotional differences, and a higher level of KOL involvement, all of which are typical characteristics of “water army” behavior that influences public opinion through unified emotional feedback. On the other hand, Cluster 1 and Cluster 2, due to their large emotional fluctuations and the higher number of KOLs, display more complex discussions and widespread emotional feedback, which typically do not align with the characteristics of “water army” behavior. Cluster 0 and Cluster 3, with more consistent emotional feedback and fewer KOLs, indicate more stable interactions with smaller emotional fluctuations, making it less likely to exhibit “water army” characteristics.

It is important to note that the high-heat comments and concentrated emotional expressions observed in the current study cannot be simply attributed to “water army” operations. In actual analysis, we found that a large number of high-interaction behaviors may stem from players who have a strong identification with the game and cultural resonance, and voluntarily express their opinions. These commenters are not hired “water army” participants but rather “emotional participants” who voluntarily engage, enthusiastically support, and actively spread content. They post comments out of emotional identification with the game’s narrative, cultural symbols, and even the development of domestic games, thereby displaying structural characteristics similar to those of water armies, such as high consistency, high interaction heat, and rapid diffusion. This is precisely where the study needs to introduce the concept of “spontaneous high-identification communities” to differentiate them from mechanical public opinion manipulation.

This type of user-driven collective expression, within the framework of sentiment evaluation theory, can be seen as an “emotional resonance phenomenon,” in which multiple users, when faced with the same cultural text, reach consistent or similar emotional judgments through similar cognitive assessment paths. From the perspective of social network theory, such emotional expressions are not “manufactured” but rather the result of “aggregation,” representing a typical decentralized opinion consensus generation mechanism.

## 5 Discussion

This study conducts a multi-dimensional data analysis of audience comments on Black Myth: Wukong, expanding the research from audience analysis to community research, covering sentiment analysis, interaction behavior, and clustering modeling. It explores the emotional responses and interaction patterns of different audience groups, combining Appraisal Theory of Emotion and Social Network Theory to reveal the role of cultural background, community structure, Key Opinion Leaders (KOLs), and potential bot behaviors in shaping user emotional expression and interaction dissemination.

Overall, user evaluations of the game show a clear positive tendency, with neutral to positive emotional feedback dominating, reflecting widespread cultural acceptance.

The cluster analysis results further reveal the complex cultural difference structures and social network dynamics behind the comments. Users from different cultural backgrounds not only show differences in comment styles but also reflect the diversity of emotional evaluation paths and cultural understanding in their interaction behaviors. These cultural interaction differences are particularly evident in the international dissemination of Black Myth: Wukong. The study found that the game, by integrating Eastern cultural elements and narrative strategies, sparked global user engagement. In Western markets, players exhibited a strong interest in Chinese traditional symbols and mythology, while domestic Chinese users found cultural identity and emotional resonance through the game.

This cross-cultural acceptance phenomenon corroborates Monceri (2022) important reflection on “cross-cultural communication as a discipline”: although this field has been integrated into the “scientific system” with its own theoretical frameworks and practical methods, its institutionalized path is still deeply influenced by Western knowledge structures. Therefore, when dealing with highly culturally embedded media practices like game dissemination, researchers should not only focus on the effects of cultural content but also examine the discourse construction mechanisms and cultural power structures behind it. As Monceri points out, cross-cultural communication research should break through the limitations of traditional Western scientific paradigms and place greater emphasis on the interactive logic and subject positioning from a multicultural perspective.

This theoretical viewpoint is validated in the audience comment analysis of this study. For example, by comparing the highest heat comments on Bilibili (a Chinese video-sharing website) and YouTube, we observe differences in how audiences perceive the same cultural content, their emotional evaluations, and interaction behaviors, showing a “cultural decoding tension” in the global context. This indicates that in the digital media environment, games not only serve as cultural products transmitting content but also as practices of cross-cultural interaction, reflecting and shaping new forms of global cultural circulation.

Furthermore, this “cultural decoding tension” is not only reflected in differences in emotional expression and interaction patterns but also reveals how platform algorithms and community rules deeply shape user behavior, raising a range of ethical concerns and platform-specific biases. Different platforms apply distinct filtering mechanisms for comment visibility, interaction prioritization, and content recommendation. For instance, YouTube tends to highlight comments with high numbers of likes, amplifying dominant emotional expressions, while Bilibili fosters stronger collective emotional resonance through real-time “bullet comments” and user-level systems, reinforcing a locally oriented cultural echo.

In this process, platform algorithms may selectively amplify certain emotional content, suppressing non-mainstream voices and creating emotional biases within the platform. These differences in “emotional visibility” can reshape the structure of opinion expression and affect how external observers interpret the overall sentiment of a user community. Moreover, some key opinion leaders (KOLs) or promotional accounts manipulate comment rankings through artificial engagement strategies—such as buying likes or coordinating fake interactions—obscuring authentic user feedback and disrupting organic emotional dissemination.

## 6 Results

This study conducts a multi-dimensional data analysis of Black Myth: Wukong audience comments, integrating sentiment evaluation, filtering threshold calculation, and cluster analysis methods. It systematically explores emotional expression, interaction behavior, and the underlying influence mechanisms within virtual game communities. The study, based on sentiment scores, measures the influence of comments using filtering thresholds (combining likes and replies) and employs K-means clustering to identify topic dimensions, opinion leader groups, and potential subgroups exhibiting bot-like behavior. Additionally, the research investigates cultural differences in game acceptance within a cross-cultural communication context, revealing significant emotional expression and identity differences between Western and Chinese users in their comments.

The results align closely with several conclusions in existing literature. For example, Social Network Theory’s concept of opinion leaders’ central role in information diffusion and Appraisal Theory’s explanation of emotional transmission pathways were empirically validated. High-centrality user comments (KOLs) significantly increased audience interaction participation, confirming a clear structural dependence of opinion formation on the community.

This study makes several important contributions:

*Methodological contribution*: For the first time, the study integrates sentiment scores, filtering thresholds, and clustering algorithms into a composite “emotion—influence—structure” model. This model provides an empirical analysis of the logical chain behind user emotional expression and interaction behavior, addressing gaps in previous research on the “complex behavior systems” in game communities.
*Theoretical contribution*: By introducing the concept of “spontaneous high-interaction player groups” as a third analytical category, this study breaks through the traditional binary judgment of “opinion leaders/bots.” It emphasizes that emotional resonance and structural expression in digital spaces can stem from natural cultural identity, national emotions, or a sense of community belonging, rather than purely from commercial manipulation. Although such collective emotional behaviors share similarities with bot-like actions, the underlying motivations differ, warranting re-modeling of their behavioral characteristics.
*Deepening cross-cultural communication theory*: Building on existing “cultural acceptance” frameworks, this study introduces a “bidirectional cultural encoding feedback mechanism.” Western users’ interest in Eastern cultural elements was found to manifest as emotional curiosity and interactive enthusiasm, while Chinese users viewed the game as an emotional outlet for national cultural pride. This interaction forms an “emotional-cultural feedback loop” that goes beyond the linear communication structure.

### 6.1 Ethical considerations

While the study successfully anonymized the data to ensure user privacy and safeguard against data breaches, it also acknowledges the ethical risks associated with web scraping and bot detection. Despite anonymization, web scraping remains ethically challenging, especially regarding the risk of unintentionally violating users’ privacy. As user comments are collected without direct consent, future research should implement stricter protocols for ethical data handling, ensuring transparency and safeguarding users’ rights during data collection.

The study also recognizes the risks of misclassification in detecting bot-like behaviors. The use of algorithms for identifying bots may inadvertently misidentify genuine user comments as automated behavior, particularly in cases of collective emotional engagement that resemble bot-like activity. In future work, it will be important to refine detection algorithms to minimize the potential for false positives in bot identification.

### 6.2 Translation and sentiment bias

Another limitation not fully addressed in this study is the sentiment analysis bias that can arise when handling translated comments or non-native English content. Many of the user comments were written by non-native English speakers or were translations, which could lead to discrepancies in emotional interpretation due to differences in cultural context or translation accuracy. To address this, the study used separate translation models tailored to specific countries and languages, improving the accuracy of sentiment analysis. However, further research should consider implementing more advanced translation quality checks or refining sentiment models to accommodate the nuances of multiple languages, ensuring a more accurate reflection of the users’ emotional states.

### 6.3 Platform algorithm considerations

Finally, the impact of YouTube’s recommendation algorithm on comment visibility and emotional amplification was not adequately discussed in this study. YouTube’s algorithm likely influences which comments are prominently displayed, amplifying dominant emotional expressions and potentially skewing the perceived sentiment within the user community. The study should consider the role of algorithmic filtering and how it affects the overall emotional dynamics on the platform. Future research could examine the relationship between algorithmic recommendations and the organic distribution of user comments to understand how platform biases influence public opinion and emotional expression within digital communities.

### 6.4 Practical implications

The findings of this study offer valuable multi-dimensional insights for game developers, community managers, and policymakers. First, identifying opinion leaders and emotional aggregation nodes can help community managers build more targeted engagement strategies, fostering stronger user participation. Second, the distinction between “organizational manipulation” vs. “spontaneous expression” provides a foundation for content moderation and public opinion regulation, helping to maintain a healthy, open, and authentic discourse environment. For global marketing strategies, understanding the significant emotional and content preference differences among users from different cultural backgrounds is crucial for tailoring communication strategies that are more locally relevant. Furthermore, the “emotion-structure-behavior” model developed in this study can inform platform recommendation algorithm optimization, ensuring more equitable information distribution and minimizing the influence of manipulated content.

### 6.5 Future research directions

Future research could explore multiple directions to deepen the findings of this study. First, introducing time series modeling and dynamic sentiment analysis can reveal the emotional evolution within the community and highlight key event-driven triggers. Second, cross-platform research using API scraping of comment and interaction data from various platforms will provide a broader understanding of cross-platform emotional migration and user behavior fluidity across different media. By combining natural language processing (NLP) and behavior tracking technologies, future studies could develop advanced bot detection algorithms to explore the underlying commercial or political motives behind bot-like behavior. Additionally, further research should analyze the psychological mechanisms of cross-cultural communication, examining how deeply cultural backgrounds influence emotional expression patterns and shaping the opinion formation and diffusion processes. Finally, integrating AI with social network analysis to develop emotion-recognition algorithms could support automated detection of KOL clusters, manipulative public opinion networks, and authentic user behavior, contributing to the sustainable and ethical development of digital communities.

## Data Availability

The datasets presented in this study can be found in online repositories. The names of the repository/repositories and accession number(s) can be found in the article/supplementary material.
