# Correction: On sentiment recognition mechanism in Black Myth: Wukong player communication on Youtube

**DOI:** 10.3389/fpsyg.2025.1729713

**Published:** 2025-11-10

**Authors:** QinLi Tang, XueJiao Bai, Feng Gan

**Affiliations:** 1 Anhui Broadcasting Movie and Television College, Hefei, China; 2 Department of Arts, International College, Krirk University, Bangkok, Thailand; 3 School of Arts, Southeast University, Nanjing, China

**Keywords:** game communities, emotional mechanism, opinion leaders and fake engagement, cross-cultural communication, user comments and behavior analysis

Affiliation Anhui Broadcasting Movie and Television College was erroneously given as Anhui University of Broadcasting and Television.

An incorrect **Funding** statement was provided. The correct funder is the Anhui Provincial Department of Education. The correct funding statement reads:

‘The author(s) declare that financial support was received for the research and/or publication of this article. This research was supported by the “Exploration and Practice of a New Teaching Paradigm for the Film and Television Program Production Major in the Context of Intelligent Media” (Project no. 2024jyxm1257). This research was supported by the Anhui Provincial Department of Education.'

The original version of this article has been updated.

